# List of Reviewers

**DOI:** 10.1002/pcn5.153

**Published:** 2023-12-26

The Editorial Board members of Psychiatry and Clinical Neurosciences Reports are grateful to the following reviewers who provided the Journal with their expertise from 1st October 2022 to 30th September 2023. Their efforts are greatly appreciated.

**Table:** 

Abe, Takaaki
Akaho, Rie
Asakura, Takashi
Baba, Hajime
Babula, Michael
Chen, Chong
Daldegan‐Bueno, Dimitri
De Winter, Lars
Ernesto, Gregorio
Farokhnezhad Afshar, Pouya
Finder, Stuart G.
Fujii, Yutaka
Fujino, Junya
Fujishiro, Hiroshige
Fujita, Junichi
Fukao, Kenjiro
Fukao, Takashi
Funatogawa, Tomoyuki
Gaetz, William
Gauthier, Serge
Hansen, Heather
Harada, Takayuki
Hashimoto, Naoki
Hata, Masahiro
Hatta, Kotaro
Hayashi, Naoki
Higashi, Shinji
Hirano, Yoji
Hori, Hikaru
Horinouchi, Toru
Iga, Junichi
Iida, Mako
Ikebuchi, Emi
Ikeda, Shunichiro
Ikenouchi, Atsuko
Ikuta,Toshikazu
Imai, Ayu
Imai, Hissei
Inada, Ken
Inagaki, Takahiko
Inoue, Shinichiro
Inoue, Takeshi
Inoue, Yushi
Ishida, Takuto
Ishida, Yasushi
Isobe, Masanori
Itagaki, Shuntaro
Ito, Kae
Ito, Yoshinori
Iwamoto, Kazuya
Iwanami, Akira
Iwata, Masaaki
Iwata, Yusuke
Jinde, Seiichiro
Jitoku, Daisuke
Kanahara, Nobuhisa
Kanemoto, Kosuke
Kasanuki, Koji
Katayama, Kohta
Katsumata, Yotaro
Katsuta, Narimasa
Kawabe, Kentaro
Kawakatsu, Shinobu
Kenzaka, Tsuneaki
Kikuchi, Akiko
Kikuchi, Yuki
Kindler, Jochen
Kishi, Taro
Kishi, Yasuhiro
Kito, Shinsuke
Ko, Chih‐Hung
Kobayashi, Ryota
Kobayashi, Toshiyuki
Kodama, Satoshi
Kondo, Shinsuke
Kondo, Tsuyoshi
Konno, Yusuke
Kosaka, Hirotaka
Kozuki, Haruka
Kubota, Manabu
Kumazaki, Tsutomu
Kuroki, Toshihide
Maia, Lucas
Markowitz, John C
Mastumoto, Yoshihiro
Matsubara, Toshio
Matsuda, Yuki
Matsui, Kentaro
Matsumoto, Takuya
Matsuo, Koji
Matsuoka, Teruyuki
Minami, Shota
Mitra, Sayantanava
Mitsuda, Dai
Miyata, Jun
Nakagami, Yukako
Nakajima, Shun
Nakamura, Katsunori
Nakamura, Masayuki
Narita, Hisashi
Narita, Zui
Nemoto, Kiyotaka
Ng, Qin Xiang
Niimura, Hidehito
Nishida, Keiichiro
Nishitani, Shota
Nishizono‐Maher, Aya
Noda, Yoshihiro
Noma, Shun'ichi
Obata, Shugo
Oda, Hiroyuki
Ogata, Haruhiko
Ogawa, Asao
Ogawa, Shintaro
Ohi, Kazutaka
Oi, Hitomi
Okajima, Yoshiro
Okamoto, Yuri
Okubo, Ryo
Ota, Miho
Ota, Toyosaku
Otowa, Kenji
Ramírez‐Bermúdez, Jesús
Sadahiro, Ryoichi
Saito, Junichi
Saito, Sinnosuke
Saito, Tamaki
Sakai, Yoshie
Sakata, Masatsugu
Sakayori, Takeshi
Sakurai, Hitoshi
Sasabayashi, Daiki
Sasayama, Daimei
Sato, Shinji
Schenck, Carlos
Seino, Hitomi
Seto, Hidefumi
Shiina, Akihiro
Shimizu, Eiji
Shimoda, Kengo
Shinagawa, Shunichiro
Shinosaki, Kazuhiro
Shiozaki, Kazumasa
Shiraishi, Nao
Shobassy, Ahmad
Shoji, Mikio
Siqueira‐Campos, Vania
Suda, Tetsufumi
Sueki, Hajime
Tachikawa, Hirokazu
Tada, Mariko
Tajika, Aran
Takagi, Shunsuke
Takahashi, Hidetoshi
Takai, Michiko
Takamura, Masahiro
Takano, Ayumi
Takebayashi, Minoru
Takechi, Hajime
Takekita, Yoshiteru
Takeshima, Tadashi
Takubo, Youji
Tanaka, Mika
Tanaka, Satoshi
Tanaka, Shinichiro
Tarumi, Ryosuke
Tateno, Masaru
Terao, Takeshi
Tesen, Hirofumi
Toda, Hiroyuki
Tominaga, Toshiyuki
Tsuboi, Takashi
Tsujii, Noa
Uchida, Megumi
Uchida, Takahito
Uchino, Takashi
Ueno, Fumihiko
Umemura, Tomotaka
Usami, Masahide
Usui, Kensuke
Vesnaver, Elisabeth
Wada, Ken
Wada, Saho
Wynn, Jonathan
Yada, Yuji
Yagyu, Kazuyori
Yamada, Hisashi
Yamada, Kazuo
Yamada, Yuto
Yamaguchi, Sosei
Yamashita, Hiroshi
Yamauchi, Tsuneo
Yasuma, Naonori
Yoshida, Naofumi
Yoshimura, Masafumi
Yoshimura, Reiji
Yoshimura, Sayaka
Yoshimura, Yuko
Yoshinaga, Naoki
Yoshino, Atsuo

